# A cluster randomized trial of Visitect CD4 Advanced Disease platform among outpatients with advanced HIV disease in Uganda

**DOI:** 10.1002/jia2.70075

**Published:** 2026-01-21

**Authors:** Elizabeth Nalintya, Elizabeth L. Schwartz, Patricia Nerima, Ann Fieberg, Sarah M. Najjuka, Ruth E. Ajilong, Tessa Adzemovic, Olive Namakula, Atukunda Mucunguzi, Kiiza Kandole Tadeo, Mark Oilor, Preethiya Sekar, Alice Lehman, Bruce Larson, Biyue Dai, David B. Meya, David R. Boulware, Radha Rajasingham, the ENCORE team

**Affiliations:** ^1^ Infectious Diseases Institute Makerere University Kampala Uganda; ^2^ Division of Infectious Diseases and International Medicine Department of Medicine University of Minnesota Minneapolis Minnesota USA; ^3^ Division of Epidemiology and Community Health School of Public Health University of Minnesota Minneapolis Minnesota USA; ^4^ Division of Biostatistics and Health Data Science School of Public Health University of Minnesota Minneapolis Minnesota USA; ^5^ University of Minnesota Medical School Minneapolis Minnesota USA; ^6^ Boston University School of Public Health Boston Massachusetts USA

**Keywords:** advanced HIV disease, opportunistic infections, point‐of‐care, CD4 count, Visitect

## Abstract

**Introduction:**

Despite significant progress in HIV care globally, a persistent 30–40% of people present with advanced HIV disease with ≤200 CD4 cells/µl. The Visitect CD4 Advanced Disease platform is a point‐of‐care CD4 test being implemented in resource‐limited settings. We sought to assess clinical outcomes of survival and retention‐in‐care among people with advanced HIV disease based on CD4 testing modality.

**Methods:**

We performed a cluster randomized clinical trial to evaluate the Visitect CD4 compared with onsite standard laboratory‐based CD4 testing. The trial was conducted at 16 outpatient HIV clinics in Uganda. Those identified with CD4≤200 cells/µl received a standardized package of care for advanced HIV disease. The primary outcome was 24‐week survival with retention‐in‐care. A costing analysis was performed. Randomization by CD4 methodology was stopped on 18 June, 2024, as the Visitect CD4 Advanced Disease platform was being implemented widely in Uganda, and randomization to non‐Visitect CD4 platforms was unethical if no alternative CD4 strategies were available in a timely manner. We conducted a micro‐costing analysis to estimate the resources used for each trial participant over the 6‐month study period.

**Results:**

Between 5 May 2022 and 18 February 2025, 1724 participants were enrolled; 927 participants received Visitect CD4 testing (eight clusters), and 797 received standard CD4 testing (eight clusters). The composite endpoint of death or lost to follow‐up occurred in 7.0% (63/901) who received Visitect CD4 testing and 7.2% (57/788) who received standard CD4 testing (hazard ratio, 0.98; 95% CI, 0.69, 1.40). The estimated risk difference between arms was 0.01% (95% CI, −2.5, 2.5). Median time to antiretroviral therapy initiation was 0 days with Visitect testing versus 7 days with standard CD4 testing (adjusted hazard ratio, 1.23; 95% CI, 1.05, 1.45). Mean cost of 6‐month care was US$115 for Visitect CD4 testing versus US$131 for standard‐of‐care CD4 testing.

**Conclusions:**

Implementation of Visitect CD4 testing demonstrated more rapid initiation of HIV therapy with equivalent 24‐week survival and retention‐in‐care compared with other point‐of‐care CD4 strategies at equivalent cost. Despite its poor specificity, the Visitect CD4 platform remains a cost‐neutral option compared to standard CD4 modalities.

**Article Summary Line:**

In this cluster randomized trial, we identified that participants with advanced HIV disease who were randomized to receive the Visitect CD4 Advanced Disease platform had equivalent 24‐week survival with retention‐in‐care compared with standard CD4 testing strategies.

## 1 INTRODUCTION

Despite significant progress in HIV prevention and treatment, a persistent 30−40% of people present with advanced HIV disease [1], defined by the World Health Organization (WHO) as having a CD4 ≤200 cells/µl or the presence of WHO Clinical Stage 3 or 4 events [2]. CD4 testing is recommended to identify individuals with advanced HIV disease to facilitate timely screening and prophylaxis for opportunistic infections. Uptake of CD4 testing remains variable in Southern and Eastern Africa [3, 4].

CD4 testing by flow cytometry has historically been the gold standard. Though highly sensitive and specific, the variable turnaround time for flow cytometry results in centralized laboratories presents a missed opportunity for early screening and prophylaxis for opportunistic infections such as tuberculosis (TB) and cryptococcosis before antiretroviral therapy (ART) initiation [5]. Point‐of‐care CD4 testing decreases time to ART initiation among people with advanced HIV [6]. Several devices for point‐of‐care CD4 testing are commercially available, including the BD FACSPresto system (BD Biosciences, Franklin Lakes, NJ, USA) and the PIMA CD4 platform (Abbott, Chicago, IL, USA). These devices require laboratory infrastructure, electricity and a steady supply of cartridges, which present logistical challenges for resource‐limited settings. Moreover, both BD Biosciences and Abbott have announced that they will no longer make new CD4 machines. Thus, the future of point‐of‐care CD4 testing remains uncertain, especially in resource‐poor settings where the burden of advanced HIV disease is the greatest.

The Visitect CD4 Advanced Disease platform (Accubio Ltd., Alva, United Kingdom) is the only commercially available semiquantitative test that indicates whether the CD4 cell count is less than or equal to 200 cells/µl [7]. It is a disposable, point‐of‐care, instrument‐free immunochromatographic assay that provides a result within 40 minutes [8]. While the sensitivity of Visitect is very high (100% in one study), its specificity is limited at 81% [9]. Clinical outcomes among people with advanced HIV disease who receive Visitect CD4 testing have not previously been evaluated, and may guide rollout and implementation of this test.

We sought to assess survival and retention‐in‐care among people with advanced HIV disease who received Visitect CD4 testing compared with standard CD4 testing. We hypothesized that testing with the Visitect CD4 Advanced Disease platform would result in improved survival with retention‐in‐care compared with other standard CD4 methodologies. A cluster randomized trial design was chosen as CD4 methodology could be practically randomized at the clinic laboratory level, not at the individual level.

## 2 METHODS

### 2.1 Trial design

We performed a 2×2 factorial cluster randomized clinical trial to evaluate:

- Visitect CD4 Advanced Disease platform compared with standard CD4 testing, and
- Enhanced screening and prophylaxis for opportunistic infections compared with the current standard‐of‐care.

Participants were adults, aged ≥18 years, with initial CD4 results ≤200 cells/µl. Exclusion criteria are listed in the Supplementary Methods. Pregnant women were eligible for participation, but teratogenic medications were withheld as needed.

Screening for the trial occurred at the point of CD4 testing. Once clinics were assigned their CD4 methodology, all persons who were newly entering or re‐entering care would receive CD4 testing. The study nurse would review CD4 logs, and all participants with a CD4 result ≤200 cells/µl were considered for participation based on the above eligibility criteria. Participants were eligible if the assigned CD4 result was ≤200 cells/µl, even if confirmatory testing was discordant.

### 2.2 Study setting and location

The ENCORE trial was conducted at 16 outpatient HIV clinics in the Kampala and Wakiso districts of Uganda. Each cluster consisted of one HIV clinic. Thirteen clinics were government‐funded public health facilities, while the remaining three consisted of one private hospital, one private not‐for‐profit organization and one non‐governmental organization clinic.

### 2.3 Interventions

The CD4 intervention consisted of Visitect CD4 Advanced Disease testing performed at the site clinic laboratory. Testing procedures are described elsewhere [9]. Visitect CD4 testing was performed on venous whole blood in clinical laboratories by a laboratory technician employed by the health facility.

Standard CD4 testing was performed by any other method that was acceptable by the Ugandan Ministry of Health. The most common methods of standard CD4 testing were the PIMA CD4 Analyser (Abbott) and BD FACSPresto (BD Biosciences).

The enhanced screening for opportunistic infections included the FujiFilm SILVAMP TB LAM (FujiLAM II) and cryptococcal antigen (CrAg) semi‐quantitative (SQ) lateral flow assay (Immy, Norman, Oklahoma, USA). Enhanced prophylaxis included 1 month of isoniazid and rifapentine (1HP) for latent TB treatment, and treatment for disseminated cryptococcal infection if plasma CrAg titre was 3+ or greater by CrAg‐SQ.

Standard‐of‐care opportunistic infection screening included the CrAg lateral flow assay (Immy), TB LAM (Alere, Waltham, MA), fluconazole for CrAg‐positive persons and 6 months of isoniazid for latent TB treatment (or 12 weeks of weekly isoniazid and rifapentine), per WHO guidelines [2].

### 2.4 Outcomes

The primary outcome of the study was 24‐week survival with retention‐in‐care among participants (measured as a dichotomous variable). Retention‐in‐care was defined as having any interaction with the original HIV clinic (medical staff, laboratory or pharmacy) within 90 days of the 24‐week scheduled visit. Lost to follow‐up was defined as not having any interaction with the HIV clinic (medical staff, laboratory or pharmacy) for >90 days. Secondary outcomes included time to CD4 results, time to opportunistic infection testing and time to ART initiation. We also measured the incidence of opportunistic infections, hospitalizations, serious adverse events (SAEs) and virologic failure at 24 weeks.

See the Supplementary Appendix for additional details regarding sample size and randomization procedures.

### 2.5 Changes to trial design

Confirmatory CD4 testing by flow cytometry was performed at a central laboratory on samples initially processed with the Visitect CD4 Advanced Disease platform, with a separate validation study now published [9]. Upon discovering the poor specificity of the Visitect CD4 test, a decision was made to present the analysis based on all enrolled participants, but additionally present the results for participants with a confirmed CD4 ≤200 cells/µl.

To ensure that the study had adequate power for assessing the enhanced screening and prophylaxis in the advanced HIV population, additional participants were enrolled in the Visitect CD4 sites to replace participants whose initial Visitect CD4 result was ≤200 cells/µl, but confirmatory CD4 results were >200 cells/µl.

Randomization by CD4 methodology was stopped on 18 June 2024, for the remaining sites for several reasons. The Visitect CD4 Advanced Disease platform was being implemented widely in Uganda, and randomization to a non‐Visitect CD4 platform was unethical if no alternative CD4 strategies were available in a timely manner. Additionally, the independent Data and Safety Monitoring Board determined that comparison between multiple point‐of‐care methodologies was no longer scientifically relevant, as the time from blood draw to receipt of CD4 results was equivalent between arms. Here, we present results from the CD4 factorial for the 16 participating clinics where CD4 testing was randomized. The evaluation of enhanced opportunistic infection screening and prophylaxis is ongoing, and results are not presented here. For details regarding trial implementation, see Supplementary Methods.

### 2.6 Statistical methods

Baseline characteristics were analysed using the Kruskal−Wallis test for continuous variables or Fisher's exact test for categorical variables. *P*‐values for the comparisons between the treatment groups were adjusted for the cluster randomized design by a two‐step approach where the summary statistic (mean for continuous variables, mean rate for categorical variables) at each of the 16 clinics was first calculated, and the summary statistic at each site was then treated as an individual data point in the *t*‐test [10]. Cox regression with mixed effects was used to compare Visitect and standard of care testing with respect to the primary endpoint of 24‐week survival with retention‐in‐care and other time‐to‐event outcomes. Risk difference between study arms was estimated using Kaplan−Meier estimators. The proportional hazard assumption was assessed via a score test. Sensitivity analyses were conducted with a generalized linear mixed model treating the 24‐week status as a binary outcome and adjusting for baseline CD4 as a covariate [11]. A resampling approach was used to estimate the intra‐cluster correlation. Administrative censoring was applied on Day 168. For the primary outcome time‐to‐event analysis, participants who were withdrawn or transferred were censored at the time of withdrawal or transfer. For the binary sensitivity analysis, participants who were censored were classified as alive and retained in care. Analyses were performed in SAS9.4 (SAS Institute Inc., Cary, NC, USA) and R Statistical Software (version 4.4.1; R Core Team 2021).

### 2.7 Costing analysis

We conducted a micro‐costing analysis to estimate the resources used for each trial participant over the 6‐month study period. For detailed methods, please see the Supplementary Methods within the Appendix.

## 3 RESULTS

**Figure 1:**
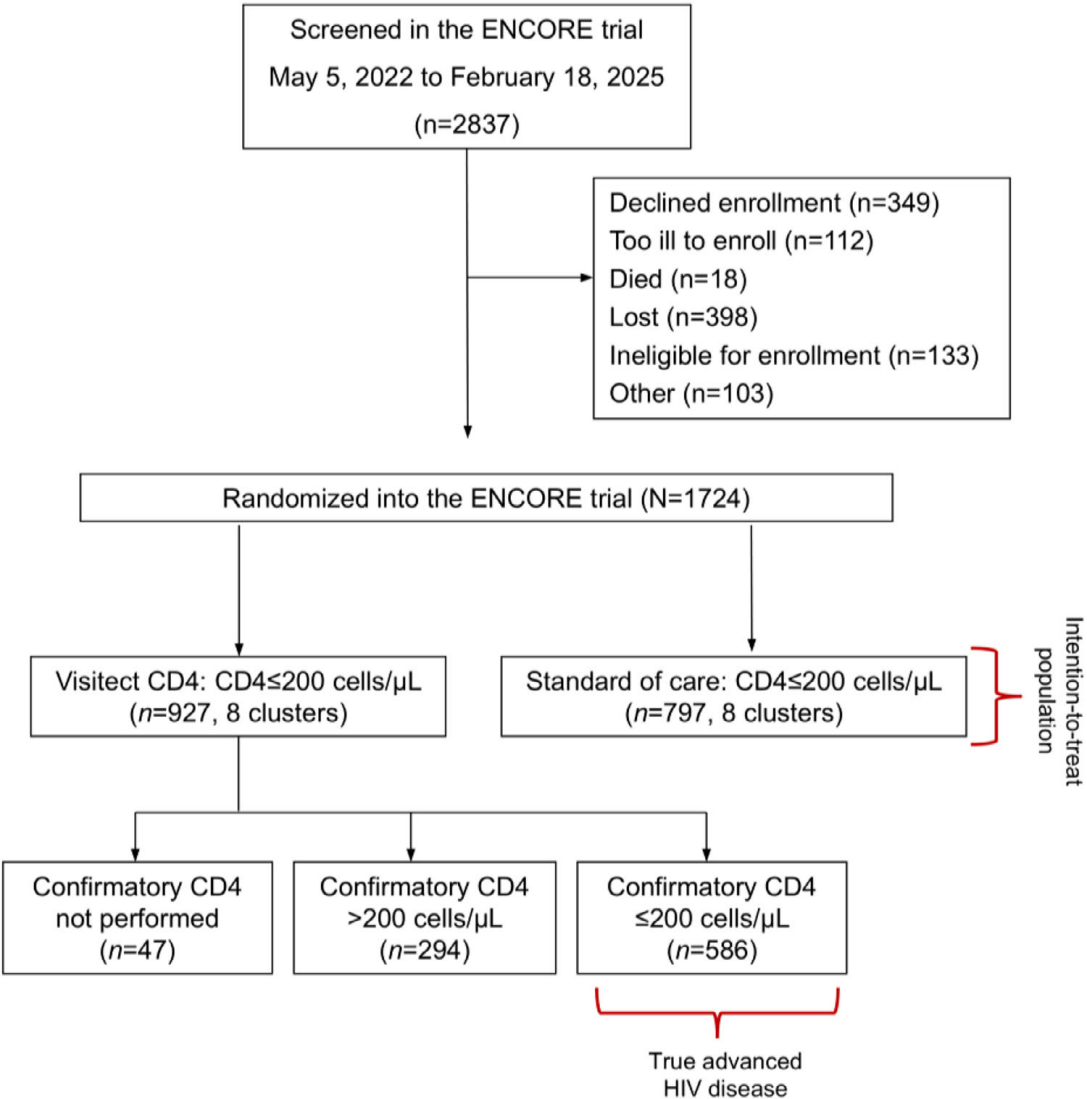
Consort diagram of screening and enrolment for the ENCORE trial. Sixteen clusters were randomized to Visitect CD4 testing versus standard‐of‐care CD4 testing. All clusters received the allocated intervention and were included in the analysis. The average size of each cluster was 100 participants. Potential participants were screened at the point of CD4 testing (which is performed for all who are entering or re‐entering HIV care). However, given the poor specificity of the Visitect CD4 test, 294 participants initially thought to have CD4≤200 cells/µl by the Visitect CD4 test were determined to have a confirmatory CD4 result >200 cells/µl. Thus, we present an intention‐to‐treat analysis and a per‐protocol analysis, where only participants in the Visitect CD4 assignment and confirmatory CD4 testing results ≤200 cells/µl were included.

Between 5 May 2022 and 18 February 2025, 2837 CD4 tests were performed at 16 clinics with CD4 test results at or below 200 cells/µl. Overall, 1724 were enrolled in the ENCORE trial and randomized to a CD4 testing strategy (Figure 1). There were 927 participants randomized to use the Visitect CD4 Advanced Disease platform and 797 randomized to the CD4 testing standard‐of‐care.

Of the 927 participants randomized to receive CD4 testing using the Visitect CD4 Advanced Disease platform, 880 (95%) had confirmatory testing performed by flow cytometry, PIMA or BD Presto. Of the 880 Visitect samples with confirmatory testing, 294 samples (33%) revealed a CD4 value >200 cells/µl by another platform. Results are presented among all participant assignments per intention‐to‐treat, and among participants with confirmed CD4 results at or below 200 cells/µl.

### 3.1 Baseline characteristics

Among the 1724 participants enrolled, the median age was 34 years, and 51% were female. The median CD4 cell count was 110 cells/µl, 56% were ART‐naive at enrolment and the median time since HIV diagnosis was 5 days (Table 1). Overall, 25% were TB LAM positive, 35% were receiving active TB treatment and 6.3% were plasma CrAg positive.

**Table 1:** Baseline characteristics of advanced HIV disease cluster‐randomized clinical trial participants

	Total enrolled	Visitect CD4 platform	Standard‐of‐care CD4 testing	*p*‐value^a^	Adjusted *p*‐value^b^
Number of enrolled participants	1724	927	797		
Baseline characteristics					
Age, years	34 [28, 40]	32 [27, 39]	34 [29, 40]	<0.0001	0.0022
Female	887 (51%)	510 (55%)	377 (47%)	0.0014	0.0039
Weight, kg	56 [50, 62]	56 [50, 64]	55 [50, 61]	0.1129	0.5240
ART status					
ART‐naïve	965 (56%)	496 (53%)	469 (59%)	0.0252	0.4468
ART‐experienced	758 (44%)	431 (46%)	327 (41%)	0.0252	0.4468
Receiving ART at enrolment	597 (35%)	340 (37%)	256 (32%)	0.0600	0.5054
Laboratory characteristics					
CD4, cells/µl	110 [49, 177]	133 [59, 271]	95 [43, 145]	<0.0001	0.0130
CRP, mg/l	4.7 [2.8, 29.3]	3.8 [2.3, 26.1]	5.8 [2.9, 35.9]	0.0028	0.3460
HIV history					
Days since HIV diagnosis	5 [1, 213]	6 [1, 365]	3 [1, 91]	0.0015	0.8972
Opportunistic infections					
Plasma CrAg positive	108 (6.3%)	47 (5%)	61 (8%)	0.0285	0.2374
Urine TB LAM positive	426 (25%)	201 (22%)	225 (28%)	0.0017	0.3720
Receiving active TB treatment	597 (35%)	323 (35%)	274 (34%)	0.8790	0.9499

*Note*: Values are Median [IQR] or *N* (%).

Abbreviations: ART, antiretroviral therapy; CrAg, cryptococcal antigen; IQR, interquartile range; TB, tuberculosis.

^a^Unadjusted for clustering by clinic: Kruskal−Wallis or Fisher's exact test was used.

^b^Adjusted for clustering by clinic: *t*‐test or Fisher's exact test was used.

When including only participants with a confirmatory CD4 result ≤200 cells/µl, participants assigned to the Visitect CD4 platform were more likely to have a lower CD4 cell count (83 vs. 95 cells/µl) (Table S1).

### 3.2 Primary outcome: 24‐week survival with retention‐in‐care

**Figure 2:**
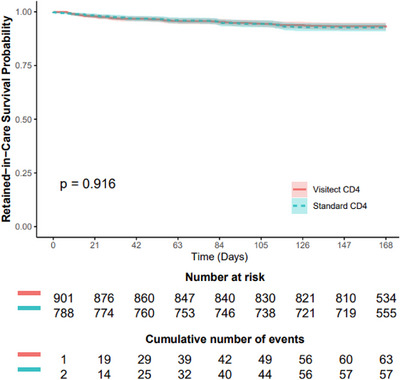
Kaplan−Meier curve of 24‐week survival with retention‐in‐care. There was no significant difference in death or lost to follow‐up between arms (hazard ratio 0.98, 95% CI, 0.69−1.40). Data are censored at 168 days. Abbreviation: CI, confidence interval.

Overall, 901 of the 927 participants assigned to Visitect CD4 testing and 788 of the 797 assigned to standard‐of‐care CD4 testing had the 24‐week outcome documented. The composite endpoint of death or lost to follow‐up occurred in 63 of 901 (7.0%) participants assigned to Visitect CD4 testing, and 57 of 788 (7.2%) assigned to standard CD4 testing. There was no significant difference in death or lost to follow‐up between arms (hazard ratio 0.98, 95% CI, 0.69−1.40) (Figure 2 and Table 2). The estimated risk difference between arms was 0·01% (95% CI, −2.5, 2.5). After including only those with confirmatory CD4 testing ≤200 cells/µl, no significant differences were found in death or lost to follow‐up between arms (hazard ratio 1.01, 95% CI, 0.68−1.51), estimated risk difference 0.14% (95% CI, −2.7 to 3.0) (Figure S1 and Table S2). The intra‐cluster correlation was 0.01 (95% CI, 0.00−0.22) in the intent‐to‐treat population, and was 0.01 (95% CI, 0.00−0.23) among participants with a confirmatory CD4 result ≤200 cells/µl.

**Table 2:** Summary of primary events by CD4 testing assignments

Outcome	Visitect CD4 platform	Standard‐of‐care CD4 testing	*p*‐value
**Randomized**	927	797	
**Week 24 outcome assessed**	901	788	
**Death or lost to follow‐up**	63 (7.0%)	57 (7.2%)	0.8473
**Death**	29	30	
**Lost to follow‐up**	34	27	
**Hazard ratio (95% CI)**	0.98 (0.69−1.40)		0.9159

Abbreviation: CI, confidence interval.

### 3.3 Secondary outcomes

**Figure 3:**
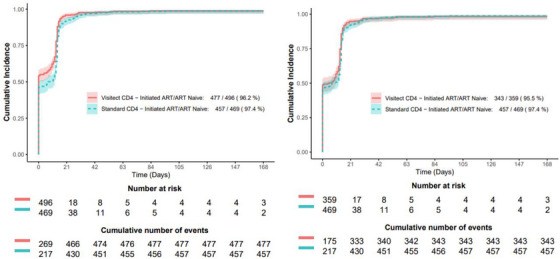
Time to ART initiation by CD4 group among participants who were ART naive. The left panel includes all participants. The right panel includes only participants with a confirmed CD4 result ≤200 cells/µl. Overall, median time to ART initiation, estimated from the Kaplan−Meier curve, was 0 days (95% CI, 0–1) among participants assigned to Visitect CD4 testing versus 7 days (95% CI, 0–12) among participants assigned to standard CD4 testing. In the Visitect CD4 arm, 54% of participants started ART on the day of enrolment; in the standard CD4 testing arm, 46% of participants started ART on the day of enrolment. Among participants with only advanced HIV disease (CD4 ≤200 cells/µl), median time to ART initiation was 5 days (95% CI, 0–10) among participants assigned to Visitect CD4 testing versus 7 days (95% CI, 0–12) among participants assigned to standard CD4 testing. Abbreviation: ART, antiretroviral therapy.

Median time to receipt of CD4 results was 0 days (IQR 0−0) among both arms. Median time from CD4 testing to receipt of opportunistic infection screening was 0 days (IQR 0−0) in both arms. Median time to ART initiation, the time when 50% of ART‐naïve participants initiated ART, was 0 days (95% CI, 0–1) among participants assigned to Visitect CD4 testing versus 7 days (95% CI, 0–12) among participants assigned to standard CD4 testing. In a mixed effect Cox model adjusted for baseline CD4, participants assigned to Visitect CD4 testing initiated ART 23% faster than participants assigned to standard CD4 testing, with an adjusted hazard ratio of 1.23 (95% CI, 1.05−1.45) in all participants and 1.18 (95% CI, 1.01−1.39) when only participants with advanced HIV disease (CD4≤200 cells/µl) were included. Time to ART initiation is summarized in Figure 3. ART was initiated on the day of enrolment among 54% assigned to Visitect CD4 testing and among 46% assigned to standard CD4 testing. However, when including only participants with advanced HIV disease (CD4≤200 cells/µl), median time to ART initiation was 5 days (95% CI, 0–10) among participants assigned to Visitect CD4 testing versus 7 days (95% CI, 0–12) among participants assigned to standard CD4 testing.

Among all participants assigned to Visitect CD4 testing, there was a trend towards fewer SAEs and hospitalizations (Table S3). However, when including only participants with a confirmatory CD4 ≤200 cells/µl, this trend was no longer present (Table S4).

Overall, 68% of participants assigned to Visitect CD4 testing and 54% of participants assigned to standard CD4 testing had a viral load measured during study follow‐up; 8.1% participants assigned to Visitect CD4 testing had virologic failure compared with 9.0% participants assigned to standard CD4 testing (*p* = 0.5971, adjusted *p* = 0.8568, Table S5).

### 3.4 Characteristics and outcomes of participants with a confirmatory CD4 >200 cells/µl

Of the 927 participants who were assigned to Visitect CD4 testing and initially identified as having a CD4 cell count ≤200 cells/µl, 294 had a confirmatory CD4 count >200 cells/µl, and 47 had no confirmatory test performed. This group with CD4 count >200 cells/µl was more likely to be female (58%) and less likely to be ART‐naive (42%) compared with participants with CD4 ≤200 cells/µl (Table S6). Median CD4 cell count in this cohort was 374 cells/µl (IQR 270–540). Median time to ART initiation for participants with a CD4 count >200 cells/µl was 0 days (95% CI, 0−0), whereas median time to ART initiation among participants with CD4 ≤200 cells/µl was 7 days (95% CI, 0–11). Only two (0.7%) were CrAg positive and, upon repeat testing, were CrAg negative. Overall, 10% were TB LAM positive, and fewer were receiving treatment for active TB (19%) compared with participants with CD4≤200 cells/µl (38%).

Among participants with a confirmatory CD4 result >200 cells/µl, significantly fewer hospitalizations occurred compared to participants with CD4 results ≤200 cells/µl (2.4% vs. 5.1%, *p* = 0.0419), and fewer opportunistic infections occurred among participants with CD4 results >200 cells/µl (26% vs. 39%, *p*<0.0001). Similarly, SAEs occurred in 3.4% with CD4>200 cells/µl versus 7.7% among participants with CD4≤200 cells/µl (*p* = 0.0089) (Table S7). Among participants with a confirmatory CD4 result >200 cells/µl, death or lost to follow‐up at 24 weeks was 6.9%, compared with 7.2% among participants with CD4 results ≤200 cells/µl (hazard ratio, 0.89 [95% CI, 0.55, 1.47]; *p* = 0.6706, Table S8).

### 3.5 Costs and resources used

Unit costs of resources used per participant are summarized by trial arm in Table S9. Mean per participant cost was $131 for standard‐of‐care CD4 testing compared with $115 for Visitect CD4 testing with confirmatory CD4 count ≤200 cells/µl (Table S10). There were no statistically significant differences in total per‐participant costs or in any individual expense category between the two study arms. Hospitalization was the largest contributor to overall costs, accounting for 29% of the total per‐participant cost in the standard‐of‐care CD4 arm and 21% in the Visitect CD4 arm among participants with a confirmatory CD4 ≤200 cells/µl.

Among participants assigned to Visitect CD4 testing whose initial CD4 results were read as ≤200 cells/µl but confirmatory CD4 testing revealed CD4>200 cells/µl, the cumulative cost of opportunistic infection diagnostics, Tuberculosis (TB) prophylaxis, fungal prophylaxis, and other laboratory tests and procedures translated to an additional $12.63 per participant with true advanced HIV disease (Figure S2). Despite this additional cost, per participant costs were equivalent between arms.

## 4 DISCUSSION

In this cluster randomized clinical trial evaluating the Visitect CD4 Advanced Disease platform in comparison with standard CD4 testing in 1724 patients entering or re‐entering HIV care in Uganda, we identified no significant difference in 24‐week survival and retention‐in‐care (hazard ratio, 0.98, 95% CI, 0.69−1.40). In the intention‐to‐treat analysis, time to ART initiation was more rapid in clinics assigned to the Visitect CD4 platform. There was a trend towards fewer severe adverse events, hospitalizations and opportunistic infections in the Visitect CD4 arm. When only analysing participants with confirmatory CD4 testing ≤200 cells/µl, time to ART was shorter among clinics assigned to the Visitect CD4 platform; however, there were no longer differences in severe adverse events, hospitalizations or opportunistic infections. Our costing analysis revealed no significant cost differences by arm, despite an additional 33% of participants without advanced HIV disease receiving opportunistic infection screening and diagnostics. Thus, despite its poor specificity, Visitect remains a cost‐neutral option compared to standard CD4 modalities.

Implementation of the Visitect CD4 intervention in this randomized trial coincided with the rollout of point‐of‐care CD4 testing by the Ugandan Ministry of Health in 2022. In 2022, the Ministry of Health phased out all flow cytometry CD4 testing and replaced it with point‐of‐care CD4 testing, including PIMA CD4 cartridge, BD FACSPresto, and even Visitect CD4 Advanced Disease platform in rural areas. This meant that the evaluation of the trial objective, evaluating the effect of Visitect CD4 testing on survival and retention‐in‐care, would be affected by this change, as now the comparison was between Visitect and other point‐of‐care tests, which were all able to provide CD4 results in a short amount of time. From the intent‐to‐treat analysis, use of the Visitect CD4 platform enabled earlier ART initiation, as the test had to be performed immediately using venous or whole blood, and the sample could not be kept overnight. With other point‐of‐care platforms, the laboratory technician can process the test on the day of blood sample collection or the following day.

The Visitect CD4 platform exhibits 93–100% sensitivity and 65–80% specificity [5, 9]. While the Visitect CD4 test is more likely to incorrectly classify a proportion of participants as advanced HIV disease whose CD4 is >200 cells/µl, survival with retention‐in‐care at 24 weeks was equivalent among the subgroup of participants with a CD4 cell count ≤200 cells/µl.

The Visitect CD4 Advanced Disease platform resulted in equivalent clinical outcomes compared with other point‐of‐care CD4 strategies at equivalent cost. The Visitect CD4 test has particular value in low‐income settings where no other CD4 modalities are available. The test could potentially be performed by lay workers outside of traditional laboratories. The other current standard CD4 tests still require electricity, cartridges and technical laboratory skills that may be unavailable in resource‐poor and rural settings; the manufacturers of these point‐of‐care tests are no longer manufacturing new machines, and, therefore, Visitect remains the only pragmatic CD4 test for settings with unstable electricity and laboratory infrastructure.

Trials assessing patient outcomes related to the implementation of Visitect testing are limited. One 2023 study from South Africa evaluated 12‐week survival with Visitect CD4 testing and an advanced HIV disease package of care [12]. Prevalence of CD4 ≤200 cells/µl was 42%, and 12‐week survival among people with advanced HIV disease was 85%, though no comparator arm was presented. In our study with WHO‐recommended screening for opportunistic infections, 24‐week survival with retention‐in‐care was 93%. With death and lost‐to‐follow‐up events occurring in one in 14 patients, screening tests to identify patients who are at high risk of death are likely warranted. One such test is the Immy CrAg lateral flow assay, which is inexpensive, easy to use and identifies people at high risk for meningitis and death. Other potential diagnostics to identify people with advanced HIV disease at high risk for poor outcomes include point‐of‐care TB diagnostics and C‐reactive protein. Such tests must be highly sensitive to serve as a triage tool and feasible in resource‐limited settings, where electricity and laboratory infrastructure may not be available.

Limitations of our study are related to the pragmatic nature of this clinical trial. The Visitect CD4 platform has imperfect specificity; thus, people without advanced HIV disease are included in our intention‐to‐treat analysis. We introduced a well‐trained research nurse to implement care for people with advanced HIV disease; this is an artificial intervention to successfully carry out the clinical trial, but likely cannot be replicated in real‐world conditions. These nurses called participants to ensure they attended clinic visits, and this likely artificially improved retention‐in‐care. Additional training was provided for laboratory technicians on performing the Visitect CD4 platform, and weekly feedback was available for each clinic. This level of quality assurance would likely not be generalizable to the real world. The test requires adherence to several timed steps, which is not always feasible in busy clinical settings. Thus, outside of clinical trial conditions, the accuracy of the Visitect CD4 platform is likely reduced, resulting in lower specificity, and more people without advanced HIV disease being incorrectly classified. Finally, the study was rolled out over 2 years, and thus, clinics that initiated later during this period may have had different characteristics compared to clinics that started in May 2023. For example, COVID‐19 outbreaks and lockdowns, Ebola outbreaks and TB screening practices may all vary with time. Theoretically, this would be accounted for after adjusting for the cluster in our analysis.

## 5 CONCLUSIONS

Point‐of‐care CD4 testing is a critical part of care for people with advanced HIV disease. Visitect CD4 Advanced Disease platform can be implemented in decentralized settings, delivers equivalent clinical outcomes compared to other point‐of‐care CD4 strategies, with shorter time to ART initiation at equivalent cost. It remains the sole point‐of‐care CD4 testing modality in resource‐limited settings.

## COMPETING INTERESTS

The authors have no conflicts of interest to declare. Visitect CD4 tests were purchased from the manufacturer.

## AUTHOR CONTRIBUTIONS

All authors have read and approved the final manuscript. EN, SMN, REA, ON, PS and AL performed the research. RR, DRB, DBM and EN designed the research study. KKT and MO assisted with laboratory training. AM, EN, ELS and PS acquired costs for the costing analysis. ELS, BL and RR performed the costing analysis. PN and AF maintained the database. AF and BD analysed the data. EN, ELS, AF, SMN, REA, MO, DBM, DRB and RR wrote the manuscript. All authors reviewed and edited the manuscript.

## FUNDING

This work was supported by the U.S. National Institutes of Health, National Institute of Allergy and Infectious Diseases (R01 AI162181, T32AI055433, K23AI138851, K24AI184270) and the Fogarty International Center (D43TW009345).

## ENCORE study team

Josephine Badaru, Faridah Nakiganda, Evelyn Natuha, Mujungu Lilian, Nuwarinda Roseline, Teopista Namuli Kateregga, Jessica Masika, Naluyima Rose, Racheal Nanono, Nakawoza Aisha Kiyemba, Jesca Asienzo, Paul Kirumira, Mathius Amperiize, Allan Buzibye, Denis Omali, Nathan Ntenkaire, Luswaata Andrew and Patience Kamarunga Mugisha.

## Supporting information




**Table S1**: Baseline characteristics among participants with confirmed CD4 ≤200 cells/µL.
**Table S2**: Summary of primary events among participants with confirmed CD4 cell count ≤200 cells/µL.
**Table S3**: Serious Adverse Event (SAE) Summary.
**Table S4**: Hospitalizations among participants with CD4 cell count ≤200 cells/µL.
**Table S5**: Summary of prevalence of virologic failure.
**Table S6**: Baseline characteristics among participants with confirmatory CD4 results >200 cells/µL.
**Table S7**: Serious Adverse Events (SAE) and Hospitalization Summary by CD4 group.
**Table S8**: Summary of primary events among participants with confirmed CD4 cell count ≤200 cells/µL compared with participants with confirmed CD4 cell counts >200 cells/µL.
**Table S9**: Unit prices by Resource Item in 2025 US dollars. All sources are per study invoices unless otherwise specified.
**Table S10**: Mean per participant costs by CD4 strategy. Costs presented in 2025 US dollars. Given the low specificity of the Visitect CD4 test, participants without advanced HIV disease were inadvertently screened for opportunistic infections and given prophylaxis. The excess costs of advanced HIV disease care among participants without advanced HIV disease was $12.63 per participant with advanced HIV disease. Despite this excess cost, costs of CD4 testing with opportunistic infection screening and prophylaxis per WHO guidelines was equivalent by arm.
**Figure S1**: Kaplan Meier curve of primary events by CD4 group, including only those with confirmatory CD4 result ≤200 cells/µL.
**Figure S2**: Per participant costs by study arm. Due to poor specificity of the Visitect CD4 platform, people without advanced HIV disease may be misclassified as having a CD4 cell count below or equal to 200 cells/µL. The cost of inappropriate opportunistic infection screening and prophylaxis among this misclassified group is on average $12.63 per participant. Despite this additional cost, mean costs per participant are equivalent between arms.

## Data Availability

Data will be openly available in Harvard Dataverse. https://doi.org/10.7910/DVN/8QPF0E.
