# Supplementary material for: A cluster randomized trial of Visitect CD4 Advanced Disease platform among outpatients with advanced HIV disease in Uganda
Source: J Int AIDS Soc. 2026 Jan 21;29(1):e70075. doi: 10.1002/jia2.70075 (PMC12824445; doi:10.1002/jia2.70075)
Supplement: Supplementary file 1 — Table S1: Baseline characteristics among participants with confirmed CD4 ≤200 cells/µL. Table S2: Summary of primary events among participants with confirmed CD4 cell count ≤200 cells/µL. Table S3: Serious Adverse Event (SAE) Summary. Table S4: Hospitalizations among participants with CD4 cell count ≤200 cells/µL. Table S5: Summary of prevalence of virologic failure. Table S6: Baseline characteristics among participants with confirmatory CD4 results >200 cells/µL. Table S7: Serious Adverse Events (SAE) and Hospitalization Summary by CD4 group. Table S8: Summary of primary events among participants with confirmed CD4 cell count ≤200 cells/µL compared with participants with confirmed CD4 cell counts >200 cells/µL. Table S9: Unit prices by Resource Item in 2025 US dollars. All sources are per study invoices unless otherwise specified. Table S10: Mean per participant costs by CD4 strategy. Costs presented in 2025 US dollars. Given the low specificity of the Visitect CD4 test, participants without advanced HIV disease were inadvertently screened for opportunistic infections and given prophylaxis. The excess costs of advanced HIV disease care among participants without advanced HIV disease was $12.63 per participant with advanced HIV disease. Despite this excess cost, costs of CD4 testing with opportunistic infection screening and prophylaxis per WHO guidelines was equivalent by arm. Figure S1: Kaplan Meier curve of primary events by CD4 group, including only those with confirmatory CD4 result ≤200 cells/µL. Figure S2: Per participant costs by study arm. Due to poor specificity of the Visitect CD4 platform, people without advanced HIV disease may be misclassified as having a CD4 cell count below or equal to 200 cells/µL. The cost of inappropriate opportunistic infection screening and prophylaxis among this misclassified group is on average $12.63 per participant. Despite this additional cost, mean costs per participant are equivalent between arms. [file JIA2-29-e70075-s001.docx]

**Supplementary Material**

Table of Contents

Supplemental Methods……………………………………………………………………...1

Supplemental Table 1……………………………………………………………………….5

Supplemental Table 2. ……………………………………………………………………...6

Supplemental Table 3. ……………………………………………………………………...7

Supplemental Table 4. ……………………………………………………………………...8

Supplemental Table 5……………………………………………………………………….9

Supplemental Table 6. ……………………………………………………………………...10

Supplemental Table 7. ……………………………………………………………………...11

Supplemental Table 8. ……………………………………………………………………...12

Supplemental Table 9. ……………………………………………………………………...13

Supplemental Table 10. ……………………………………………………………….…....15

Supplemental Figure 1. ………………………………………………………….……….....16

Supplemental Figure 2. ……………………………………………………….………….…17

References……………………………………………………………………………………….….18

Supplemental Methods

*Randomization*

The four factorial randomization assignments were 1) Visitect CD4+standard of care screening, 2) Visitect CD4 + enhanced screening, 3) standard CD4 testing + enhanced screening and 4) standard CD4 testing+ standard-of-care screening. A confidential randomization schedule with clinic numbers (1-16) and randomization assignments was generated prior to the study launch, using uniform distribution and stratified by stages. Clinics were assigned the next sequential clinic number (1-16) when ready to begin screening; if a batch of clinics onboarded simultaneously, clinic numbers were randomly assigned with unique seed for each wave. After a clinic was assigned, the randomization assignment was no longer concealed due to the open-label nature of the study. The study statistician at the University of Minnesota performed the randomization using SAS V9·4.

*Sample size*

The target sample size was 2400 persons with advanced HIV disease (CD4 ≤200 cells/µL) over a four-year enrollment period, with 24 clinics randomized in 1:1:1:1 ratio into four factorial arms and at least 100 persons enrolled in each clinic. We assumed equal cluster size with minimum enrollment in the sample size calculation. With a two-sided alpha of 0.025 to account for multiplicity for the co-primary objectives, an assumed intra-clinic correlation of 0.020 and 80% 24-week survival in the standard-of-care arm, the target sample size provides 80% power to detect a 7.9% absolute difference for the co-primary objectives. The sample size calculation was not powered to detect interactions between the factorial design’s co-primary objectives.

*Trial Implementation*

Of the 16 clinics included in this analysis, 8 enrolled from May 2022 to July 2023, and 8 enrolled from May 2023 to October 2024, with one site continuing enrollment through February 2025. One research nurse was assigned to each clinic to identify potential participants, consent and enroll participants, and ensure study interventions were performed. Specifically, the research nurse reviewed all people who had CD4 testing performed at the clinic and identified those with CD4 testing results below or equal to 200 cells/µL. If the potential participant consented to enrollment, baseline characteristics, HIV history, and current signs and symptoms of opportunistic infections were documented.

Existing health facility staff were trained to support clinical review, laboratory testing and dispensing of study medication for participants with advanced HIV disease. The study did not hire an independent team of clinical staff except for one research nurse per clinic. Opportunistic infection screening was coordinated and ensured by the research nurse, but laboratory testing (cryptococcal screening and TB LAM) was performed by laboratory technicians.

Research nurses followed participants over the 24-week study period, and documented adverse events, new hospitalizations, opportunistic infections, and final clinical outcomes. Study visits at enrollment, weeks 1, 2, 4, and 8 were performed in person. Study visits at weeks 12, 16, 20, and 24 could be performed in person or by telephone as needed. The research nurse did not call participants to remind them of their study appointments beyond the week 8 visit. However, phone calls were made at the end of the visit window for purposes of collection of vital status. Research nurses were not responsible for clinical care beyond opportunistic infection screening. The facility medical officer assessed each participant and made determinations about their presenting syndrome, further evaluation, and treatments. The research nurse documented these clinical assessments. The facility clinical team received training on advanced HIV disease, opportunistic infections, and potential study-related procedures and adverse events.

Laboratory technicians received additional training to perform the Visitect CD4 testing and screening for opportunistic infections. These training sessions were followed by monthly support supervision in the first two months of study implementation. Visitect performance data was summarized weekly and if a clinic was noted to have a high proportion of false results after confirmatory testing (>20% incongruent results), additional laboratory training was provided.

*Costing Analysis*

We conducted a micro-costing analysis to estimate the resources used for each trial participant over the six-month study period. Outpatient cost categories included: CD4 testing, opportunistic infection diagnostics and prophylaxis, laboratory tests, procedures (lumbar punctures), ART, HIV viral load, healthcare worker salaries, and laboratory technician training.

Despite this being an outpatient study, we captured inpatient costs for participants who were hospitalized. This included diagnostics, supplies, imaging, procedures, medications, lab monitoring, hospital personnel salaries, and hospital stay charges. Participant-specific hospital duration was used to estimate staff salaries, supplies, and hospital stay charges. For four key hospitalization types (TB, TB meningitis, cryptococcal meningitis, and drug-induced liver injury), we created standardized resource packages of diagnostics, imaging, procedures, medications, and lab monitoring based on clinical guidelines and trial data. For example, participants with cryptococcal meningitis were each assumed to receive one diagnostic lumbar puncture with cerebrospinal fluid (CSF) analysis, two therapeutic lumbar punctures, liposomal amphotericin B, flucytosine, fluconazole, intravenous fluids, electrolyte supplementation, and blood laboratory tests. For hospitalizations due to other causes, medical records were reviewed to estimate resource use for diagnostics, procedures, imaging, medications, and lab monitoring.

Unit costs for diagnostics, procedures, imaging, medications, and lab monitoring were obtained from trial invoices and the Global Fund Price Reference Report.[1] Overhead costs for services such as laboratory and pharmacy were incorporated into unit costs. All costs were reported in 2025 US dollars from the perspective of the healthcare payer.

Participant costs were summarized by category and compared across CD4 strategies: standard-of-care CD4 testing versus Visitect CD4 platform with confirmed results ≤200 cells/μL. Mean differences in per participant costs were estimated using linear regression with clustering at the site level. To calculate 95% confidence intervals around the mean differences, we used a wild cluster bootstrap t-test with 2,000 repetitions.

The additional cost incurred due to false-positive results from Visitect CD4 screening was also estimated. Specifically, we accounted for unnecessary interventions provided to participants with a Visitect CD4 result suggesting CD4 ≤200 cells/μL but who were later confirmed to have CD4 >200 cells/µL (N=294). We summed the costs of opportunistic infection diagnostics, TB prophylaxis, fungal prophylaxis, and other laboratory tests and procedures across these 294 participants and divided the total by the number of participants who were truly eligible for these interventions (N=586 with confirmatory CD4 ≤200 cells/µL). The costing analysis was conducted in Stata (version 18·0; StataCorp, College Station, TX).

Institutional Review Board approval was obtained through the Infectious Diseases Institute in Uganda and the University of Minnesota, USA. Approval was obtained from the Uganda National Council for Science and Technology. A waiver of consent was obtained for CD4 testing as a standard-of-care procedure; however, written informed consent was obtained from participants prior to trial enrollment to collect 6-month outcomes.

Supplemental Table 1. Baseline characteristics among participants with confirmed CD4≤200 cells/µL.

|  | Visitect CD4 platform | Standard-of-care CD4 testing | P-value^1^ | Adjusted P-value^2^ |
| --- | --- | --- | --- | --- |
| Number of enrolled participants | 586 | 797 |  |  |
| Baseline characteristics |  |  |  |  |
| Age, years | 34 [28,40] | 34 [29,40] | 0.0131 | 0.0201 |
| Female | 311 (53%) | 377 (47%) | 0.0386 | 0.0282 |
| Weight, kg | 55 [49, 62] | 55 [50, 61] | 0.6587 | 0.6932 |
| HIV history |  |  |  |  |
| Time since HIV diagnosis, days | 4 [1, 213] | 3 [1, 91] | 0.1893 | 0.6781 |
| ART naïve | 359 (61%) | 469 (59%) | 0.4049 | 0.9355 |
| ART experienced | 227 (39%) | 327 (41%) | 0.4049 | 0.9355 |
| Receiving ART at enrollment | 165 (28%) | 256 (32%) | 0.1103 | 0.7722 |
| Laboratory Values |  |  |  |  |
| CD4 (cells/µL) | 83 [34, 133] | 95 [43, 145] | 0.0084 | 0.0420 |
| CRP (mg/L) | 5·8 [2·9, 38·8] | 5·8 [2·9, 35·9] | 0.7565 | 0.5517 |
| Opportunistic Infections |  |  |  |  |
| Plasma CrAg positive | 41 (7%) | 61 (8%) | 0.6781 | 0.9492 |
| Clinical Concern for meningitis | 28 (5%) | 57 (7%) | 0.0710 | 0.5902 |
| Urine TB LAM positive | 158 (27%) | 225 (28%) | 0.6269 | 0.7549 |
| Receiving active TB treatment | 250 (43%) | 274 (34%) | 0.0020 | 0.3445 |

Median [IQR] or N (%). 1. Unadjusted for clustering by clinic: Kruskal-Wallis or Fisher's exact test were used. 2. Adjusted for clustering by clinic: T-test or Fisher's exact test were used. Clinical concern for meningitis was based on clinician assessment of symptoms. Abbreviations: ART, antiretroviral therapy; CrAg, cryptococcal antigen; TB, tuberculosis.

Supplemental Table 2. Summary of primary events among participants with confirmed CD4 cell count ≤200 cells/µL.
Abbreviation: CI, confidence intervals.

|  | Visitect CD4 platform | Standard-of-care CD4 testing | P-value |
| --- | --- | --- | --- |
| Randomized | 586 | 797 |  |
| Week 24 outcomes assessed | 570 | 788 |  |
| Death or lost to follow-up | 41 (7.2%) | 57 (7.2%) | 0.9774 |
| Death | 22 | 30 |  |
| Lost to follow-up | 19 | 27 |  |
| Hazard ratio (95%Cl) | 1.01 (0.68, 1.51) | | 0.9374 |

| Supplemental Table 3. Serious Adverse Event (SAE) Summary | | | | |
| --- | --- | --- | --- | --- |
|  | **Visitect CD4 platform** | **Standard-of- care CD4 testing** | **P-value**^1^ | **Adjusted P-value**^2^ |
| Randomized | 927 | 797 |  |  |
| Number with SAE | 57 (6.1%) | 63 (7.9%) | 0.1533 | 0.0812 |
| Total number of SAEs | 58 | 69 |  |  |
| Suspected opportunistic Infection | 34 (58.6%) | 46 (66.7%) |  |  |
| **Seriousness** |  |  |  |  |
| Hospitalization | 36 (62.1%) | 51 (73.9%) |  |  |
| Immediately life threatening | 21 (36.2%) | 18 (26.1%) |  |  |
| Other | 2 (3.4%) | 1 (1.4%) |  |  |
| N (%) Expected SAEs | 57 (98.3%) | 69 (100.0%) |  |  |
| **Relation to Opportunistic infection prophylaxis** |  |  |  |  |
| Not related | 55 (94.8%) | 68 (98.6%) |  |  |
| Possibly related | 2 (3.4%) | 0 (0.0%) |  |  |
| Probably related | 1 (1.7%) | 1 (1.4%) |  |  |
| Definitely related | 0 (0%) | 0 (0%) |  |  |
| **Outcome** |  |  |  |  |
| Recovered or resolved | 6 (10.3%) | 1 (1.4%) |  |  |
| Death | 28 (49.1%) | 29 (42.0%) |  |  |
| Clinically stable, expect to resolve | 24 (41.4%) | 37 (53.6%) |  |  |
| Unknown | 0 (0%) | 2 (3%) |  |  |
| Missing | 0 (0%) | 0 (0%) |  |  |

Values are N (%). 1. Unadjusted for clustering by clinic: Chi-square test p-value . 2. Adjusted for clustering by clinic: Chi-square test p-value. Abbreviation: IQR, interquartile range.

Supplemental Table 4. Hospitalizations among participants with CD4 cell count ≤200 cells/µL

|  | **Visitect CD4 platform** | **Standard CD4 testing** | **P-value** | **Adjusted P- value** |
| --- | --- | --- | --- | --- |
| Randomized | 586 | 797 |  |  |
| Number Hospitalized | 25 (4.3%) | 46 (5.8%) | 0.2102 | 0.1532 |
| Total number of hospitalizations | 24 | 51 |  |  |
| Suspected opportunistic infection | 14 (56.0%) | 38 (74.5%) |  |  |
| Expected | 24 (96.0%) | 51 (100.0%) |  |  |
| **Relationship to Opportunistic infection prophylaxis** | |  |  |  |
| Not related | 24 (96.0%) | 50 (98.0%) |  |  |
| Possibly related | 0 (0.0%) | 0 (0.0%) |  |  |
| Probably related | 1 (4.0%) | 1 (2.0%) |  |  |
| Definitely related | 0 (0.0%) | 0 (0.0%) |  |  |
| **Outcome** |  |  |  |  |
| Recovered or resolved | 5 (20.0%) | 1 (2.0%) |  |  |
| Death | 5 (20.0%) | 12 (23.5%) |  |  |
| Clinically stable, expected to resolve | 15 (60.0%) | 36 (70.6%) |  |  |
| Unknown | 0 (0.0%) | 2 (3.9%) |  |  |

Adjusted p-value is from Fisher’s Exact Test or Chi-Square Test for categorical variables.

Supplemental Table 5. Summary of prevalence of virologic failure.

|  | **Visitect CD4 platform** | **Standard CD4 testing** | **P-value** | **Adjusted P-value** |
| --- | --- | --- | --- | --- |
| Participants randomized | 927 | 797 |  |  |
| Participants with a viral load after Day 7 | 632 (68.2%) | 434 (54.5%) |  |  |
| Participants with virologic failure  (Viral load >400 copies/mL) | 51 (8.1%) | 39 (9.0%) | 0.5971 | 0.8568 |

N(%); P-values are calculated with a chi-squared test. Adjusted P-values are calculated with a two-step approach to account for clustering

Supplemental Table 6. Baseline characteristics among participants with confirmatory CD4 results >200 cells/µL

|  | CD4<200 cells/µL | CD4>200 cells/µL | All enrolled |
| --- | --- | --- | --- |
| Number of enrolled participants | 1383 | 294 | 1724 |
| Baseline characteristics |  |  |  |
| Age, years | 34 [28, 40] | 30 [25, 37] | 34 [28, 40] |
| Female | 688 (49.7%) | 170 (57.8%) | 887 (51.5%) |
| Weight, kg | 55 [49, 61] | 58 [52, 66] | 56 [50, 62] |
| HIV history |  |  |  |
| Time since HIV diagnosis, days | 4 [1, 152] | 8 [1, 365] | 5 [1, 213] |
| ART naïve | 828 (60%) | 124 (42%) | 965 (56%) |
| ART experienced | 554 (40%) | 170 (58%) | 758 (44%) |
| Receiving ART at enrollment | 421 (31%) | 148 (50%) | 596 (35%) |
| Laboratory values |  |  |  |
| CD4 (cells/µL) | 89 [38, 141] | 374 [270, 540] | 110 [49,177] |
| CRP (mg/L) | 5.8 [2.9, 36.6] | 2.9 [1.2, 7.0] | 4.7 [2.8, 29.3] |
| Opportunistic infections |  |  |  |
| Plasma CrAg positive | 102 (7.4%) | 0 (0.0%) | 108 (6.3%) |
| Clinical concern for meningitis | 85 (6.1%) | 6 (2.0%) | 93 (5.4%) |
| Urine TB LAM positive | 383 (27.7%) | 29 (9.9%) | 426 (24.7%) |
| Receiving active TB treatment | 524 (37.9%) | 57 (19.4%) | 597 (34.6%) |

Median [IQR] or N(%). Abbreviations: ART, antiretroviral therapy; CrAg, cryptococcal antigen; IQR, interquartile range; TB, tuberculosis.

| Supplemental Table 7. Serious Adverse Events (SAE) and Hospitalization Summary by CD4 group. | | | |
| --- | --- | --- | --- |
|  | **CD4 <200 cells/µL** | **CD4 >200 cells/µL** | **P-value** |
| Randomized | 1383 | 294 |  |
| Number with SAE | 106 (7.7%) | 10 (3.4%) | 0.0089 |
| Total number of SAEs | 112 | 11 |  |
| Suspected opportunistic infection | 72 (64.3%) | 5 (45.5%) |  |
| **SAE seriousness** |  |  |  |
| Hospitalization | 76 (67.9%) | 7 (63.6%) |  |
| Immediately life threatening | 36 (32.1%) | 3 (27.3%) |  |
| Other | 2 (1.8%) | 1 (9.1%) |  |
| N (%) Expected SAEs | 111 (99.1%) | 11 (100.0%) |  |
| **Outcome of SAE** |  |  |  |
| Recovered or Resolved | 6 (5.4%) | 0 (0.0%) |  |
| Death | 51 (45.5%) | 6 (54.5%) |  |
| Clinically stable, expect to resolve | 53 (47.3%) | 5 (45.5%) |  |

Supplemental Table 8. Summary of primary events among participants with confirmed CD4 cell count ≤200 cells/µL compared with participants with confirmed CD4 cell counts >200 cells/µL.

|  | | **CD4 ≤200 cells/µL** | **CD4 >200**  **cells/µL** | | **P-value** |
| --- | --- | --- | --- | --- | --- |
| Randomized | | 1383 | 294 | |  |
| Week 24 outcome assessed | | 1358 | 291 | |  |
| **Death or Lost to Follow-up** | | 98 (7.2%) | 20 (6.9%) | | 0.8365 |
| Death | | 52 | 7 | |  |
| Lost to Follow-up | | 46 | 13 | |  |
| Hazard Ratio (95% CI) | 0.89 (0.55, 1.47) | | | 0.6706 | |
| Lost to Follow-up is considered as a failure event for the primary event. P-value is calculated with Chi-squared test, without adjusting for clustering. Abbreviation: CI, confidence interval. | | | | | |

Supplemental Table 9. Unit prices by Resource Item in 2025 US dollars. All sources are per study invoices unless otherwise specified.

| **Resource Category** | **Resource Item** | **Unit Price (2025 USD)** | **Source** |
| --- | --- | --- | --- |
|  | Visitect CD4 Advanced Disease Platform  (Accubio Ltd. Alva, United Kingdom) | 3.98 | ^1^ |
| **CD4 and viral load testing** | BD FACSPresto CD4 System  (BD Biosciences, Franklin Lakes, NJ, USA) | 8.57 | ^1^ |
|  | BD FACSCount CD4 Flow Cytometry  (BD Biosciences, Franklin Lakes, NJ, USA) | 5.85 | ^1^ |
|  | PIMA CD4 Platform  (Abbott, Chicago, IL, USA) | 3.80 | ^1^ |
|  | HIV Viral Load | 9.66 | ^1^ |
| **Opportunistic Infection Diagnostics** | Acid-Fast Bacillus Smear | 7.00 |  |
|  | Acid-Fast Bacillus Culture | 45.00 |  |
|  | Xpert MTB/RIF Assay  (Cepheid, Sunnyvale, CA, USA) | 9.98 | ^1^ |
|  | Xpert MTB/RIF Ultra Assay  (Cepheid, Sunnyvale, CA, USA) | 14.90 | ^1^ |
|  | Determine TB LAM  (Alere, Waltham, MA) | 3.50 | ^1^ |
|  | FujiFilm SILVAMP TB LAM | 6.00 | ^2^ |
|  | Cryptococcal Antigen (CrAg) Lateral Flow Assay (Immy, Norman, Oklahoma, USA) | 3.00 |  |
|  | Cryptococcal Antigen Semiquantitative (CrAgSQ) Lateral Flow Assay  (Immy, Norman, Oklahoma, USA) | 1.30 |  |
| **Other Laboratory Tests** | CSF Analysis (not including CrAg) | 9.52 |  |
|  | Creatinine | 6.80 |  |
|  | Alanine Aminotransferase (ALT) | 6.80 |  |
|  | Bilirubin | 6.80 |  |
|  | Potassium | 5.44 |  |
|  | Magnesium | 5.44 |  |
|  | Complete Blood Count | 4.08 |  |
|  | Pregnancy Test | 0.02 |  |
| **Medications** | Dolutegravir + Lamivudine + Tenofovir fixed-dose combination tablet | 0.12 | ^1^ |
|  | Isoniazid 300mg + Rifapentine 300mg tablet | 0.40 | ^1^ |
|  | Rifapentine 150mg tablet | 0.25 | ^1^ |
|  | Isoniazid 300mg tablet | 0.02 | ^1^ |
|  | Pyridoxine 50mg tablet | 0.02 |  |
| **Inpatient Medications** | Amphotericin B deoxycholate 50mg vial | 8.57 |  |
|  | Flucytosine 500mg tablet | 5.10 |  |
|  | Fluconazole 200mg tablet | 0.21 |  |
|  | Ethambutol 275mg + Isoniazid 75mg + Pyrazinamide 400mg + Rifampin 150mg (RHZE) -fixed-dose combination tablet | 0.06 | ^1^ |
|  | Isoniazid 150mg + Rifampin 300mg fixed dose tablet | 0.04 | ^1^ |
|  | Liposomal Amphotericin B 50mg vial | 16.25 | ^3^ |
|  | Magnesium supplementation 143mg tablet | 0.14 |  |
|  | Potassium supplementation 20mEq tablet | 0.21 |  |
|  | IV fluids 500mL | 0.41 |  |
|  | Ceftriaxone 2g | 4.76 |  |
| **Other** | Hospital Stay -1 day | 5.37 | ^4^ |
| **Imaging & Procedures** | Abdominal ultrasound | 13.60 |  |
|  | Chest X-ray | 13.60 |  |
|  | Head CT | 65.30 |  |
|  | Abdominal CT | 65.30 |  |
|  | Upper Endoscopy | 81.63 |  |
| **Lumbar puncture supplies** | Spinal needle | 1.05 |  |
|  | Falcon tube -15mL | 0.21 |  |
|  | Cryovial | 0.15 |  |
|  | Syringe -2mL | 0.07 |  |
|  | Lidocaine 1mL | 0.02 |  |
| **General Hospital Supplies** | Butterfly Needle | 0.99 |  |
|  | Sterile Gloves | 0.30 |  |
|  | Vacutainer needle | 0.14 |  |
|  | IV tubing | 0.14 |  |
|  | Latex Gloves | 0.11 |  |
|  | Gauze Pad | 0.09 |  |
|  | Alcohol Swab | 0.01 |  |
|  | Band-aid | 0.01 |  |
| **Monthly salaries of Personnel** | Physician | 1564.53 |  |
|  | Laboratory technician | 1306.04 |  |
|  | Phlebotomist | 1306.04 |  |
|  | Nurse | 1265.23 |  |
|  | HIV Counselor | 1115.58 |  |

Supplemental Table 10. Mean per participant costs by CD4 strategy. Costs presented in 2025 US dollars. Given the low specificity of the Visitect CD4 test, participants without advanced HIV disease were inadvertently screened for opportunistic infections and given prophylaxis. The excess costs of advanced HIV disease care among participants without advanced HIV disease was $12.63 per participant with advanced HIV disease. Despite this excess cost, costs of CD4 testing with opportunistic infection screening and prophylaxis per WHO guidelines was equivalent by arm.

| Expense category | Standard CD4 testing (USD)  n=797 | Visitect CD4, confirmed CD4≤200 cells/µL (USD)  n=586 | Mean Difference | 95% Confidence Interval |
| --- | --- | --- | --- | --- |
| CD4 testing | 5.52 | 3.98 | -1.54 | (-3.72, 0.32) |
| Opportunistic infection screening diagnostics | 9.92 | 9.84 | -0.08 | (-2.65, 2.74) |
| TB prophylaxis | 10.46 | 9.39 | -1.07 | (-7.56, 5.53) |
| Fungal prophylaxis | 16.95 | 15.82 | -1.13 | (-18.16, 16.05) |
| Outpatient labs & procedures | 4.74 | 3.67 | -1.07 | (-6.33, 4.08) |
| ART | 18.60 | 17.85 | -0.75 | (-1.51, 0.84 |
| HIV Viral load | 5.95 | 7.78 | 1.83 | (-0.94, 4.81) |
| Personnel salaries & training | 21.65 | 22.59 | 0.94 | (-0.46, 2.21) |
| Hospitalization | 37.47 | 23.99 | -13.48 | (-34.45, 9.63) |
| Total | **131.27** | **114.92** | **-16.35** | **(-48.17, 18.69)** |
| Total plus additional cost of participants with confirmatory CD4>200 cells/µL | **131.27** | **127.55** | **-3.72** | **(-35.54, 31.32)** |

Supplemental Figure 1. Kaplan Meier curve of primary events by CD4 group, including only those with confirmatory CD4 result ≤200 cells/µL.


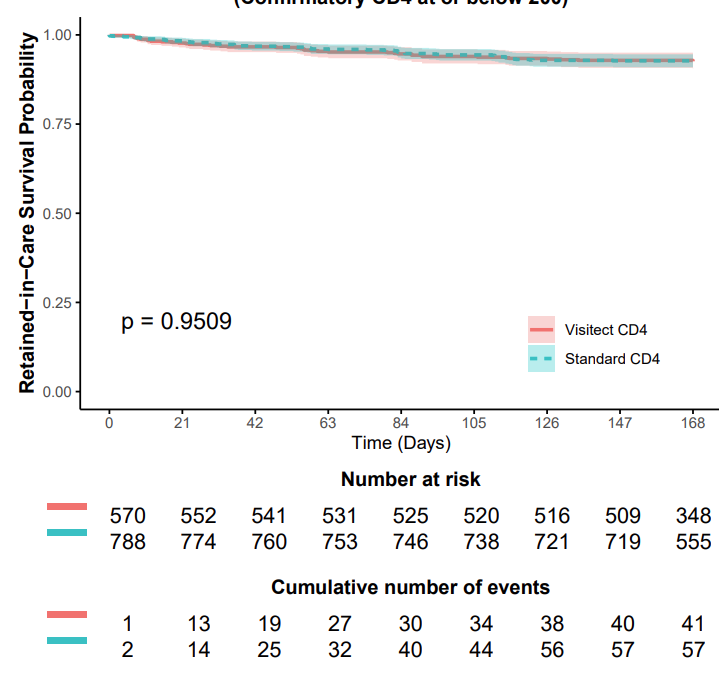


Supplemental Figure 2. Per participant costs by study arm. Due to poor specificity of the Visitect CD4 platform, people without advanced HIV disease may be misclassified as having a CD4 cell count below or equal to 200 cells/µL. The cost of inappropriate opportunistic infection screening and prophylaxis among this misclassified group is on average $12.63 per participant. Despite this additional cost, mean costs per participant are equivalent between arms.


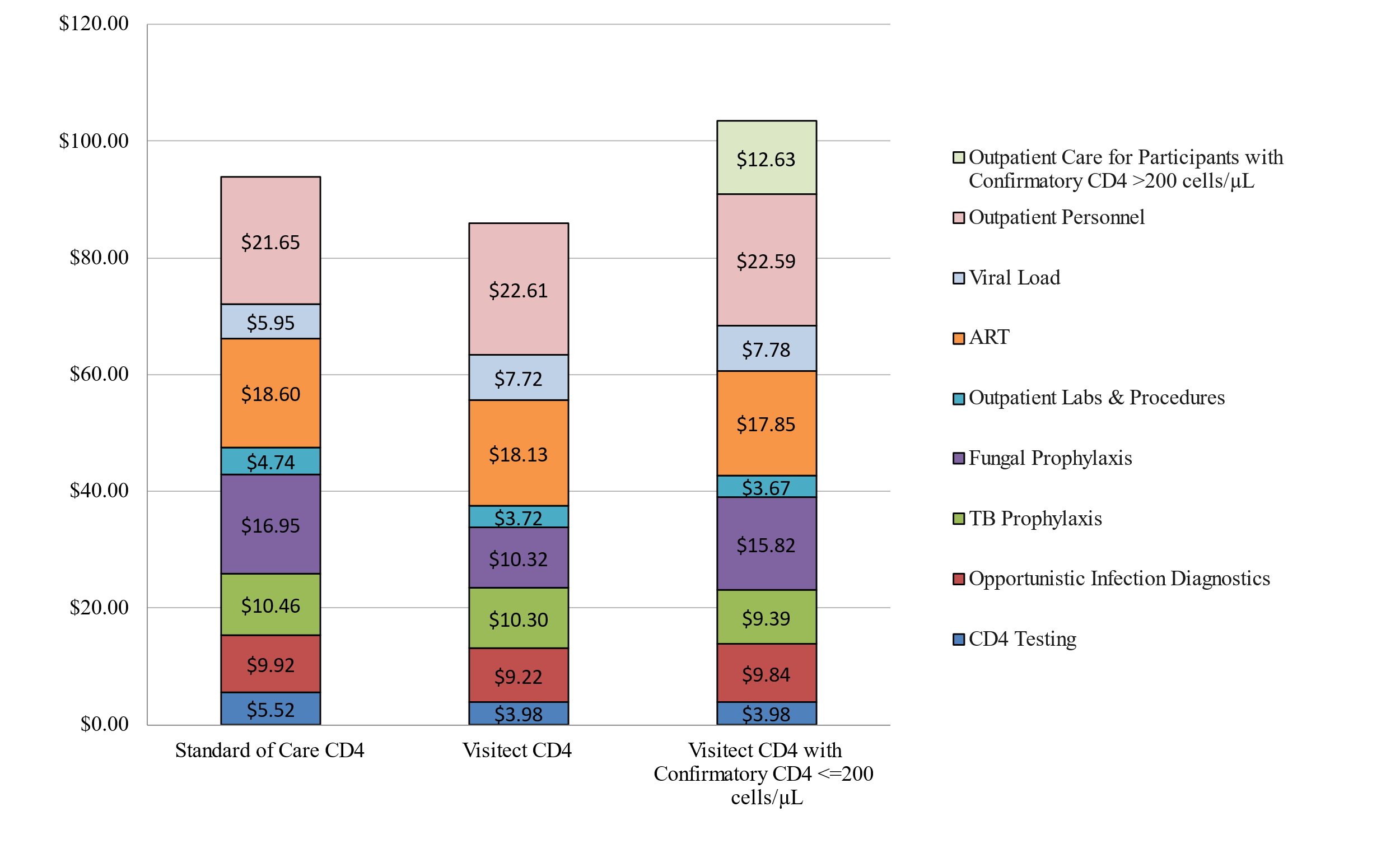


References

1. The Global Fund,. Price and Quality Reporting [Internet]. 2023 Oct. Available from: https://www.theglobalfund.org/en/sourcing-management/price-quality-reporting/

2. Brümmer LE, Thompson RR, Malhotra A, Shrestha S, Kendall EA, Andrews JR, Phillips P, Nahid P, Cattamanchi A, Marx FM, Denkinger CM, Dowdy DW. Cost-effectiveness of Low-complexity Screening Tests in Community-based Case-finding for Tuberculosis. Clin Infect Dis Off Publ Infect Dis Soc Am. 2024 Jan 25;78(1):154–163. PMCID: PMC10810711

3. Rajasingham R, Nalintya E, Israelski DM, Meya DB, Larson BA, Boulware DR. Cost-effectiveness of single-dose AmBisome pre-emptive treatment for the prevention of cryptococcal meningitis in African low and middle-income countries. Med Mycol. 2022 Feb 1;60(2):myab078. PMCID: PMC9756002

4. Rajasingham R, Rolfes MA, Birkenkamp KE, Meya DB, Boulware DR. Cryptococcal meningitis treatment strategies in resource-limited settings: a cost-effectiveness analysis. PLoS Med. 2012;9(9):e1001316. PMCID: PMC3463510
